# Supplementary material for: Determination of Dapoxetine Hydrochloride in Human Plasma by HPLC–MS/MS and Its Application in a Bioequivalence Study
Source: Molecules. 2022 Apr 22;27(9):2707. doi: 10.3390/molecules27092707 (PMC9101379; doi:10.3390/molecules27092707)
Supplement: Supplementary file 1 [file molecules-27-02707-s001.zip › molecules-1692065-supplementary.pdf]

Supplementary Materials

Determination of Dapoxetine Hydrochloride in Human Plasma by HPLC–MS/MS and Its Application in a Bioequivalence Study

Xin Zhang [1], Zhanwang Gao [1], Fei Qin [2], Kehan Chen [1], Jiansong Wang [2,*] and Lingli Wang [1,*]

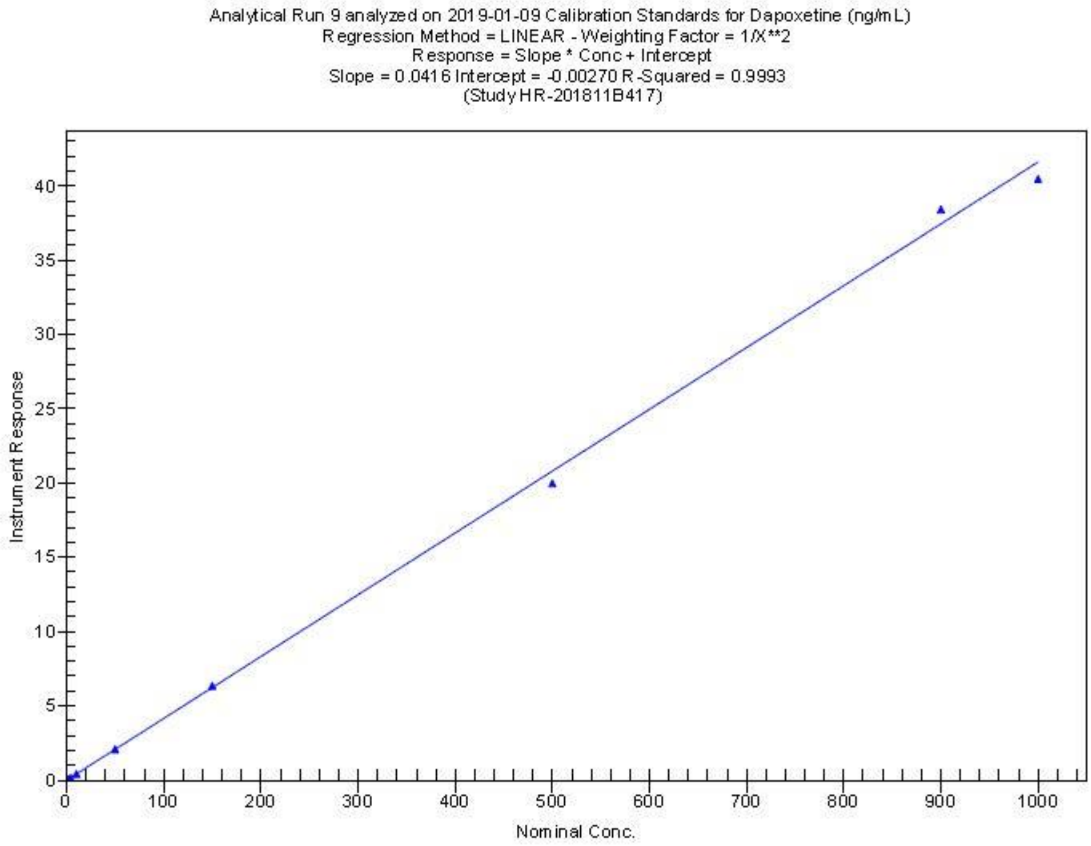

Figure S1. The standard curve.

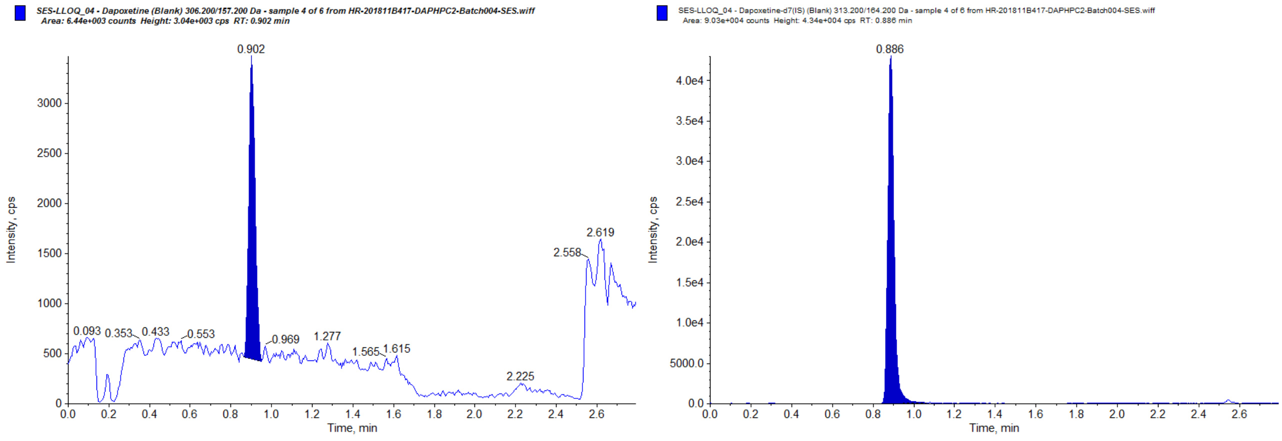

Figure S2. The chromatogram of analyte and IS.
